# A Tractable Method for Measuring Nanomaterial Risk Using Bayesian Networks

**DOI:** 10.1186/s11671-016-1724-y

**Published:** 2016-11-15

**Authors:** Finbarr Murphy, Barry Sheehan, Martin Mullins, Hans Bouwmeester, Hans J. P. Marvin, Yamine Bouzembrak, Anna L. Costa, Rasel Das, Vicki Stone, Syed A. M. Tofail

**Affiliations:** 1 Kemmy Business School, University of Limerick, Limerick, Ireland; 2 RIKILT Wageningen University & Research (WR), Akkermaalsbos 2, 6708 PD Wageningen, The Netherlands; 3 Division of Toxicology, Wageningen University, Stippeneng 4, 6708 WE Wageningen, The Netherlands; 4 ISTEC-CNR, Via Granarolo, 64, I-48018 Faenza, RA Italy; 5 Nanotechnology and Catalysis Research Center, University of Malaya, 50603 Kuala Lumpur, Malaysia; 6 Heriot-Watt University, Edinburgh, EH14 4AS Scotland, UK; 7 Department of Physics, and Bernal Institute, University of Limerick, Limerick, Ireland

**Keywords:** Risk assessment, Control banding, Bayesian

## Abstract

While control banding has been identified as a suitable framework for the evaluation and the determination of potential human health risks associated with exposure to nanomaterials (NMs), the approach currently lacks any implementation that enjoys widespread support. Large inconsistencies in characterisation data, toxicological measurements and exposure scenarios make it difficult to map and compare the risk associated with NMs based on physicochemical data, concentration and exposure route. Here we demonstrate the use of Bayesian networks as a reliable tool for NM risk estimation. This tool is tractable, accessible and scalable. Most importantly, it captures a broad span of data types, from complete, high quality data sets through to data sets with missing data and/or values with a relatively high spread of probability distribution. The tool is able to learn iteratively in order to further refine forecasts as the quality of data available improves. We demonstrate how this risk measurement approach works on NMs with varying degrees of risk potential, namely, carbon nanotubes, silver and titanium dioxide. The results afford even non-experts an accurate picture of the occupational risk probabilities associated with these NMs and, in doing so, demonstrated how NM risk can be evaluated into a tractable, quantitative risk comparator.

## Background

Control banding has been identified [1, 2] as a suitable framework in which concerned stakeholders can evaluate and determine the risks of nanomaterials (NMs) to human health. Despite a broad consensus on the approach, there are no significant implementations that have a widespread support. This is because of known [3–5] inconsistencies in characterisation, toxicological measurement and exposure tests that are especially difficult to map using a risk tool. The low volume of quality data relevant to NM risk measurements presents further difficulties in the hazard potential. Widespread recognition that the hazard potentials of NMs can vary depending on chemistry, physicochemical characteristics, concentration and the mode and time of exposure.

Several unique control banding (CB) solutions have been identified [6] that utilize exposure and hazard banding to evaluate occupational risk for nanomaterials. Each CB tool offers alternative methodologies in order to estimate the exposure potential and hazard, and hence classify the prevailing risk within a ranking matrix. For example, NanoSafer [7] combines a hazard evaluation derived from data provided by technical information sheets, with an exposure assessment determined by the occupational setting and production rates to provide case-specific risk assessment of manufactured nanomaterials. Similarly, the Nano-Evaluris [8] CB solution assesses occupational inhalation risk pertaining to nanopowders by combining hazard and exposure band estimates with protective measures taken, process emission evaluation and frequency of use. The physical form (solid, liquid, powder or aerosol) of a manufactured nanomaterial is used to determine the exposure/emission potential within the French agency for food, environmental, and occupational health and safety (ANSES) [9] CB framework, with the hazard band allocated according to the classification of the bulk or analogous material according to the classification, labelling and packaging (CLP) regulation. While CB tools are effective in classifying risk and establishing risk management protocols 6, current applications have been criticised regarding their inability to produce transparent or easily communicable risk forecasts. Alternative frameworks employ multi criteria decision analysis (MCDA) and weight of evidence (WoE) approaches, which enable expert judgement and experimental data to be incorporated into the risk assessment [10–12]. Indeed, for regulatory purposes, MCDA-based tools may provide a more appropriate way to address issues surrounding data uncertainty [13, 14]. However, the uncertainties underlying the use of expert opinion to interpret the quality of experimental evidence or to establish weights for criterion in MCDA approaches may prove to have a critical effect on the resultant assessment [11]. Finally, methodologies borrowing from the finance industry [15], value of information and portfolio decision analysis can also be employed with some success.

Bayesian networks (BNs) can be utilised to overcome these limitations. BNs offer a reliable method for NM risk estimation owing to their ability to capture data sets that have a probability distribution of values or even missing values. These omissions are commonly observed in toxicological investigations related to NM risk characterisation and assessment. Furthermore, BNs allow for the incorporation of expert opinion where data are lacking, and has the functionality to refurbish these assessments as new experimental data becomes available. The model can incorporate NM-specific physicochemical characteristics, exposure potential and hazard components relevant to NMs for all exposure routes. In this article, we demonstrate the use of BN in occupational settings (e.g. inhalation exposure). The risk estimation is transparent and can be used to prioritize further testing to increase accuracy. More detailed NM characterisation, toxicological and exposure information will produce a more accurate risk estimation.

This framework enables proactive, iterative risk assessment through its underlying Bayesian interpretation of probability. Probability is subjective representing a degree of belief that is updated as information or data is acquired. In scarce data environments, new experimental data and relevant data from literature to have a strong influence on posterior probabilities as the process is updated via the learning algorithm. This, we believe, is a strength over the existing quantitative MCDA approaches [11] as the uncertainty attributed to expert opinion diminishes as additional experimental data are acquired. The margin of exposure (or MOE) of a NM is the ratio of its no-observed-adverse-effect level (NOAEL) to its predicted dose. Within a BN, the MoE can then be mapped to a control banding framework that affords even non-experts an accurate picture of the risk profile associated with a particular NM. Furthermore, the value of the risk forecast is easily translated into a decision-making framework by applying the US Environmental Protection Agency (USEPA)-defined [16] inflection point of 1 (or 100% in percentage terms) to determine whether a specific NM poses a hazard to human health.

## Methods

We apply our methodology to NMs made from silver (Ag), titanium dioxide (TiO_2_) and carbon nanotubes (CNTs) and focus on occupational exposure. For all three NMs, we source data from the US National Institute for Occupational Safety and Health (NIOSH) exposure recommendation reports [17–19] and EU funded research consortia [20–22] and then map the estimated risk to a control banding solution using our model. To test the validity of our approach, we run the model using a sample set of data from a European research consortium database [23].

BNs are a class of probabilistic models originating from the Bayesian statistics and decision theory combined with graph theory [24, 25], which are able to model dependencies between variables. They were developed as a probabilistic structure in 1921 for the analysis of crop failure [26] and re-invented by many researchers under numerous pseudo-names such as *causal network*, *belief network* and *influence diagram* [27]. Modern applications of BNs are used in the fields of medicine [28], information technology and engineering [29], food fraud prediction [30] and environmental and human health risk assessment [31–36].

**Fig. 1:**
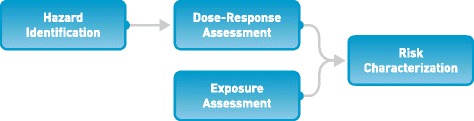
The 4-step top-down human health risk assessment framework

BNs offer an adaptive risk evaluation framework on two separate levels. First, the model structure and parameterization can be refined as contemporary research grows and improves the underlying assumptions used in the preliminary model formulations. Second, BNs are easily updatable as new scientific data becomes available by means of learning and updating model parameters and probability distributions via Bayes’ theorem [37]. The ability to incorporate a variety of traditional (i.e. experimental data) and non-traditional knowledge bases such as expert judgement, mechanistic or physical relationships and simulated data into the parameterization process of a BN appeals to the task of modelling complex systems in data-scarce environments, such as the NM risk assessment arena [38]. Using BNs, a generalised risk assessment model (Fig. 1) can be followed for the purpose of risk characterization of potentially hazardous substances and then applied to a control banding framework.

The transferability of the BN modelling framework to the NM risk assessment paradigm such as control banding is characterised by its mathematical flexibility in terms of probability elicitation (mechanical, empirical or expert judgement), its ability to adapt to new information and its capacity to produce probabilistic forecasts in low, and often missing data environments. Any variable (node) in the graphical structure may have any number of parents, including no parents. However, by increasing the number of parents with multiple states conditionally linked to a specific node, the number of conditional probabilities or parameters, to be estimated grows exponentially. While this may pose an issue as the structure grows in complexity, there are strategies to approximate the conditional probability tables which have proven adequate for these instances in the form of noisy probability distributions [39]. For example, there is a widespread uncertainty in the identification of hazard, or more precisely, in determining the lowest threshold for hazard identification. In applying BN, we resort to *expert elicitation* from both primary (SANOWORK http://cordis.europa.eu/project/rcn/102461_en.html) and secondary (NIOSH) data to ascertain input parameters and the influence thereof on predecessors and successors in the causal network leading to risk characterisation. Expert interpretations in terms of the potential for human health risk [40] are then incorporated in a BN where the uncertainty of risk-associated variables are described as probability distributions [41].

In line with Bergamaschi et al. (2015) [42], the following NM characteristics were selected as model parameters that contribute to hazard: size, surface area, chemical reactivity, surface charge, solubility and morphology. These were selected following an appraised of the literature and selected based on their influence in the particle’s toxicological profile. They [*ibid*] also detail the hazard or effect consequence of each physicochemical characteristic. We also categorize NMs as carcinogenic, mutagenic or toxic for reproduction (CMR) [43]. The choice of physicochemical characteristics is based on extant literature but of course there is no clear consensus within that literature set. BNs, however, can be configured to accommodate diverse user opinions on specific hazard impacts and multiple other parameters although a consensus or default assumption would make the prediction better.

For toxicants, a threshold exposure level can be derived, below which it is assumed there is no statistically significant adverse effect to human health. As a point of departure for this, the no-observed-adverse-effect level (NOAEL) is usually derived in animal studies, then a safety or uncertainty factor is applied (usually 100) to determine the dose considered safe for humans. In the absence of an experimentally determined NOAEL, the quantity lowest-observed-adverse-effect level (LOAEL) can be used as is the lowest dose tested for a potential hazard. Alternatively, the benchmark dose (BMD) [44, 45] method can be employed. We will use the term *occupational exposure limit* (OEL) term as the upper boundary on acceptable dose concentrations. NIOSH periodically disseminate new scientific data relating to potential occupational hazard from NMs (such as scientifically derived NOAELs) and recommend occupational exposure limits. There is, however, considerable disparity between recommended OELs proposed by NIOSH, regulatory bodies and academia. For example, current recommendations relating to CNTs range from 1 to 50 μm/m^3^ [17]. NMs’ human occupational exposure potential is the probabilistic measure of the propensity of the NM to enter the human body by inhalation, ingestion or dermal pathways. For NMs, there remains substantial ambiguity as regards the most relevant exposure metric [4, 46–48]. This remains a challenge as only a limited number of the nanomaterial parameters can be determined reliably [4, 49]. We have used the expectation–maximization (EM) learning algorithm in the BN to refine the conditional probabilities in parameterization of the risk assessment model using literature and experimental data. We also demonstrate the learning ability of BNs to handle risk data for CNT, Ag and TiO_2_ NMs as new scientific data and/or expert knowledge becomes available. For example, recommended OELs and occupational exposure data provided in the NIOSH reports [17–19] are used to learn the parameters within their corresponding nodes.

Within each node of the BN, we incorporate *experience* to measure the confidence attributed to the conditional probabilities inferred via specialist data sources. Any initial subjective choices of data can be refined to reduce bias using more data from diverse sources. This learning-through-experience feature allows us to continually define the sensitivity of the models’ parameters to new information. The idea is that if a given expert (or a group of experts) has a low confidence in their initial data choice, new information would make a considerable impact on this initial estimate and support the subsequent data with better confidence and *experience*. Once the model is updated with case data, the experience value corresponding to each state within a node coincides with the number of cases that have been observed.

## Results

**Fig. 2:**
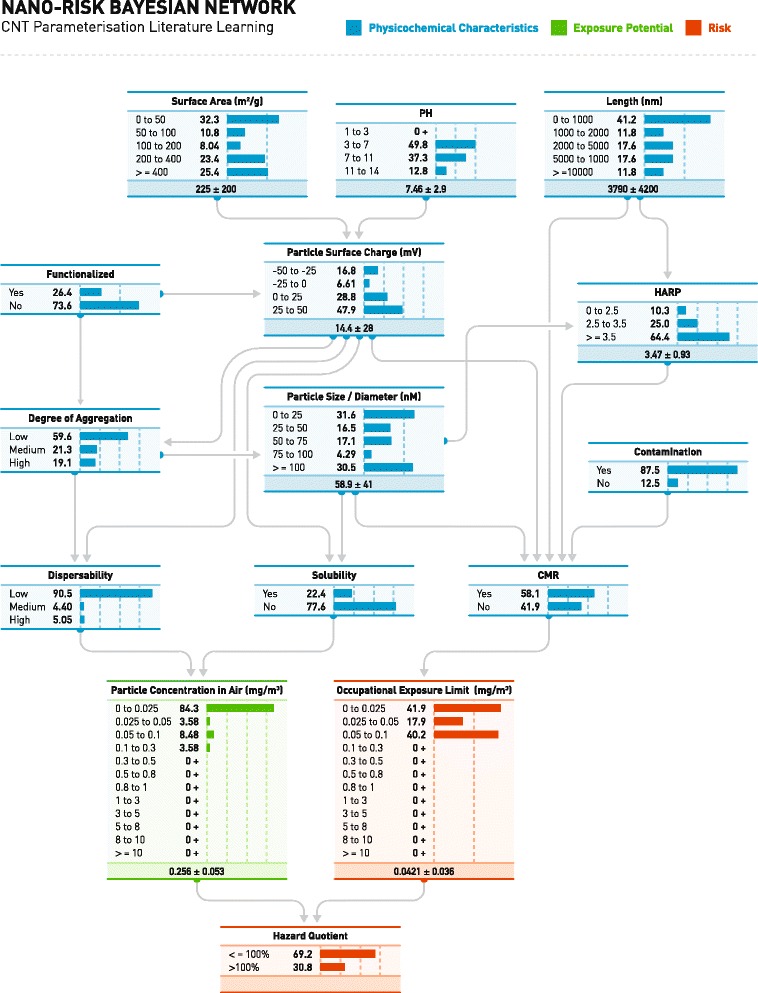
Graphical structure and parameterization for the physicochemical characteristics component of the CNT Bayesian network. Each node displays the variable name (*top*), possible states (*left*) and the % probability of being in a specific state inferred from the conditional probability table for the node with associated bar chart (*centre, right*). *Directed arrows* symbolise the conditional relationship between parent and child nodes. Continuous variables display the centre of the probability distribution and its variance. The parameter data was sourced from the National Institute for Occupational Safety and Health (NIOSH) and the EU Project, SANOWORK

Figure 2 summarises the 12 key physicochemical characteristics identified by us as influential variables for defining the potential exposure and toxicity of pristine CNT; the figures for Ag and TiO_2_ are available as supplementary data. This includes the discretized states and causal links between these states. The presence of a surface coating is also included as it is experimentally proven to induce changes in the state of specific structural determinants of hazard when compared to an uncoated, pristine state [42]. The degree of agglomeration/aggregation and dispersibility are additional characteristics known to be important factors in the causal chain of assigning NMs risk potential [40]. Conditional relationships, signified by directed arrows starting from the influential parent nodes and ending at the child nodes, are determined by expert opinions derived from relevant contemporary literature [17–22] and SANOWORK [32, 40]. For example, the directed arrow from *Degree of Aggregation* to *Particle Size* implies that the rate at which NMs attach to other NMs of the same type has a direct impact on the NM’s size distribution. Marginal variables (i.e. the nodes with no network parents, (*Coating, pH* and *Contamination*)) are assumed to be represented by uniform distributions for unbiased parameterization when adequate knowledge is lacking [32].

The exposure potential component is conditionally linked to the physicochemical characteristics component via parent nodes *dispersibility* and *solubility* (Fig. 2). The relationship is inferred from the expert opinion (NIOSH reports) used in developing the influence diagram [40]. The *NMs Concentration in Air* variable is discretized into intervals that gradually increase in range as the value of the variable increases to offer increased granularity at the left tail of the distribution where the likelihood of occurrence is greater.

Both exposure potential and dose-response assessments are incorporated in the BNs to generate a quantitative forecast of risk with parameterization (Fig. 2). Two distinct dose-response assessment models have been tested for carcinogenic (linear model) and non-carcinogenic (threshold model) toxicants [41, 50] via the OEL and are directly parameterized by its parent node *CMR*. Integrating both of these dose-response assessment ideologies into the Bayesian framework enables us to create a risk assessment model that accounts for both inter-batch and experimental inconsistencies in the relevant data, and also captures the uncertainty surrounding the toxicity potential of many NMs [51].

We define the margin of exposure as the hazard quotient (HQ), quantified by dividing the exposure potential of NMs concentration in air by the threshold value OEL. The resultant value represents the initial deterministic risk forecast generated by the BN risk assessment model. If the forecasted exposure level is greater than the threshold dose, *i.e*. HQ > 100%, there exists potential risks of adverse human health implications for the particular NM. The HQ can be refined through a robust learning process in BN and offers a coherent and quantifiable human health risk assessment. It consolidates exposure, hazard and dose-response assessments into a single risk forecast.

## Discussion

**Fig. 3:**
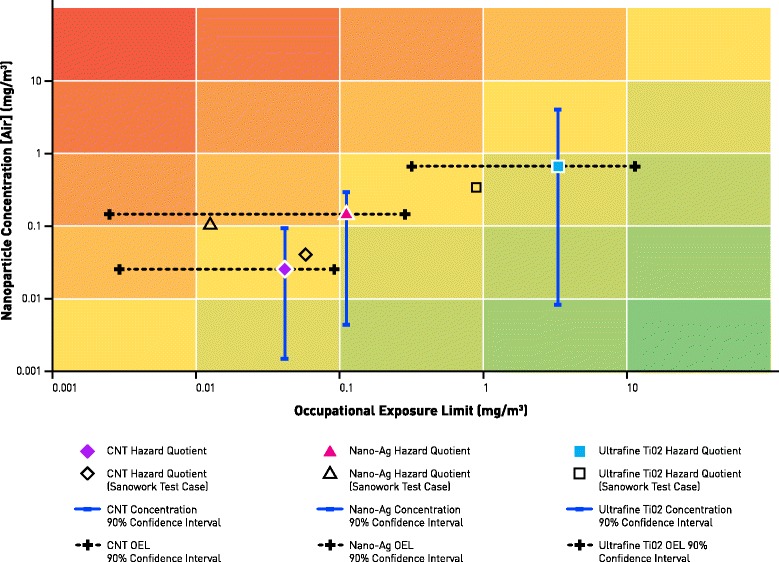
Control banding results for CNTs, Ag and TiO2. The *dashed line* represents the occupational exposure limit, and the *solid line* depicts NM concentration, both at the 90% confidence level. HQ coordinates are determined by the estimated mean values of NOAEL and NM

For CNTs, Ag and TiO_2_ NMs, we created a database of information based on the NIOSH and EU research reports on each material [17–22]. Each NIOSH report referenced numerous publications from which the secondary data was sourced. We created 46 rows of CNT data, 39 rows of Ag data and 55 rows of TiO_2_ data. All references and data are available as supplementary data. The datasets contained high levels of missing data as is typical of data from different sources. The results are plotted in a heat map (Fig. 3) and show the estimated risk of each material using the BN approach.

At a glance, we can see from Fig. 3 that CNTs, Ag and TiO_2_ NMs exhibit increasing levels of risk as we would anticipate. The heat map can be superimposed onto a control banding solution that could exhibit hazard and exposure rows and columns, this may be particularly suitable for labelling purposes or a decision-making process. The relatively wide confidence bands are indicative of the amount and quality of the available data. Clearly, more data sources with less missing data will strengthen the degree of certainty.

To further validate our approach, we then applied the same BN approach to a primary data source, the SANOWORK database. This is, in effect, an out-of-sample test to check the accuracy of the *learned* system. We include these results in Fig. 3. The results from the secondary and primary data are consistent with expectations with the out-of-sample results falling within the 90% confidence interval.

## Conclusions

Our BN approach has empirically found a solution to measuring the risk of NMs which has been a particularly vexing problem across the lifecycle of manufactured NMs. In this article, we have used secondary data from research articles cited by the NIOSH occupational exposure reports and EU research consortia to estimate the risk of CNTs, Ag and TiO_2_ and to map these results to a control banding solution that is intuitive and accessible to a wide variety of interested parties. The approach identified here is applied with a particular bias on human health factors, particularly in an occupational setting but there is no reason not to extend the approach to include a more general human health and environmental risks. To be sure, a greater discussion surrounding the input parameters and causal relationships is inevitable, but if a consensus can be found then, by definition, we have a template for a database design that can be used by experts in categorization, exposure assessment and toxicology. Where more data from research and industry published to this template, then the accuracy of risk measurement would quickly increase.

Our approach is a quantitative solution that offers a more objective approach than subjective, semi-quantitative methods. In addition to offering an alternative in the continuum of risk modelling approaches (e.g. mechanistic, statistical, Bayesian and decision-analytic [13]) for NM risk, our BN tool could also be used in conjunction with a weight of evidence [10] approach and/or multi criteria decision analysis methodologies [52]. With a quantitative result, users will be in a position to reduce exposure pathways or ameliorate hazard profiles by engineering the NM through coating (say). This BN approach is a particularly powerful approach when combined with material modelling and safety by design paradigms. By modelling the physicochemical properties of proposed NMs, an estimate of the potential risk increase/reduction of the resultant material can be derived. It may also be developed as a tool to promote occupational safety standards and extended to examine all NM lifecycle risks. With this said, the strength of BN approach derives from a consensus view on the variables and a resultant standardised database. The corollary of disperse, heterogeneous datasets with a wide variability in material properties will limit the potential of BN techniques. Therefore, national and supranational efforts to standardise nanomaterial information sets are highly desirable.

## Abbreviations

BMD: Benchmark dose
BN: Bayesian Networks
CB: Control Banding
CLP: Classification, Labelling and Packaging
CMR: Carcinogenic, Mutagenic, or toxic for Reproduction
CNT: carbon nanotube
EM: Expectation–Maximization
HQ: Hazard Quotient
MCDA: Multi Criteria decision Analysis
MoE: Margin of Exposure
NIOSH: National Institute for Occupational Safety and Health
NM: Nanomaterial
NOAEL: No Observed Adverse Effect Level
OEL: Occupational Exposure Limit
USEPA: US Environmental Protection Agency
WoE: Weight of Evidence
